# Identifying the Divergent Rural Training Needs Expressed by Older Adults and Gerontological Providers

**DOI:** 10.1093/geroni/igaf122.4134

**Published:** 2025-12-31

**Authors:** Rachel Coleman, Lenard Kaye, Susan Wehry, Mary DeSilva, Lynette Sirois, Ruth Dufresne, Katie Keough, Patricia Oh

**Affiliations:** University of Maine Center on Aging, Orono, Maine, United States; University of Maine, orono, Maine, United States; University of New England College of Osteopathic Medicine, Portland, Maine, United States; University of New England, Portland, Maine, United States; University of New England, Portland, Maine, United States; Center for Excellence in Public Health, University of New England, Portland, Maine, United States; University of New England, Portland, Maine, United States; University of Maine, Bangor, Maine, United States

## Abstract

Needs assessments of aging-related training and education commonly rely on the perspectives of those with specialized gerontological and geriatrics expertise and less so on the views of older adults themselves. Maine’s Geriatrics Workforce Enhancement Program (AgingME2), now in its second operational cycle, conducts an annual assessment of preferred training content and delivery formats identified by both older adults and gerontological professionals. The 2025 assessment (n = 254; 53.9% older adults/community members and 85.4% gerontological professionals/caregivers; multiple roles could be selected) is representative of all 16 counties in the state, the most rural in the nation. Substantial proportions of both groups (approximately one-third) indicated awareness of both the 4Ms of age-friendly health care and the age positivity movement and 32.3% and 33.6% of older adults and health care providers, respectively, have discussed 4M’s related questions during their doctor/patient interactions. Significantly larger proportions of gerontological professionals and providers prefer in-person training compared to older adults (67.4% vs. 51.8%) as well as asynchronous webinars and other brief video formats. Providers emphasize the value of technology for reducing isolation/loneliness while older adults value it for lifelong learning and locating health resources. Exercise, remaining in the community, and driving safely are prioritized by older adults while providers emphasize end-of-life, cognitive decline and polypharmacy training. There is consensus that webinars of 30-60 minutes are preferable when offered. The implications of these differing perspectives for planning gerontological education and training learning sessions for older adults compared to professional providers are considered.

